# RANKL biology: bone metabolism, the immune system, and beyond

**DOI:** 10.1186/s41232-019-0111-3

**Published:** 2020-02-07

**Authors:** Takehito Ono, Mikihito Hayashi, Fumiyuki Sasaki, Tomoki Nakashima

**Affiliations:** 1 Department of Cell Signaling, Graduate School of Medical and Dental Sciences, Tokyo Medical and Dental University, Yushima 1-5-45, Bunkyo-ku, Tokyo, 113-8549 Japan; 2 Japan Agency for Medical Research and Development, Core Research for Evolutional Science and Technology (AMED-CREST), Yushima 1-5-45, Bunkyo-ku, Tokyo, 113-8549 Japan

**Keywords:** RANKL, RANK, OPG, Bone, Immune system, Osteoimmunology, Organ development, Tumor

## Abstract

Receptor activator of NF-κB (RANK) ligand (RANKL) induces the differentiation of monocyte/macrophage–lineage cells into the bone–resorbing cells called osteoclasts. Because abnormalities in RANKL, its signaling receptor RANK, or decoy receptor osteoprotegerin (OPG) lead to bone diseases such as osteopetrosis, the RANKL/RANK/OPG system is essential for bone resorption. RANKL was first discovered as a T cell-derived activator of dendritic cells (DCs) and has many functions in the immune system, including organogenesis, cellular development. The essentiality of RANKL in the bone and the immune systems lies at the root of the field of “osteoimmunology.” Furthermore, this cytokine functions beyond the domains of bone metabolism and the immune system, e.g., mammary gland and hair follicle formation, body temperature regulation, muscle metabolism, and tumor development. In this review, we will summarize the current understanding of the functions of the RANKL/RANK/OPG system in biological processes.

## Background

The original identification of the RANKL/RANK/OPG triad took place in the late 1990s [1]. Receptor activator of NF-κB (RANK) ligand (RANKL) and its receptor RANK were discovered in the field of immunology [2]. In the first report, a novel cytokine of the tumor necrosis factor (TNF) family was shown to be highly expressed in T cells in response to T cell receptor (TCR) signaling, and was termed tumor necrosis factor (TNF)-related activation-induced cytokine (TRANCE) [3]. At almost the same time, another group cloned the receptor gene using a human dendritic cell (DC) cDNA library and its ligand, using a cDNA library of a murine thymoma cell line. In this study, the pair of the ligand and its receptor was designated as RANKL and RANK. The authors demonstrated that RANK expression is induced on differentiated CD4^+^ T cells and CD40 ligand (CD40L)-stimulated mature DCs, and that RANKL stimulation enhances T cell proliferation and T cell-DC interaction [4]. Both RANKL and RANK were shown to be crucial for the development of osteoclasts and the lymph nodes (LNs) [5, 6].

Osteoprotegerin (OPG) and osteoclastogenesis inhibitory factor (OCIF) were discovered as the result of the search for osteoclastogenesis–inhibiting factors, and later turned out to be the same molecule [7, 8]. Soon after the discovery, binding partners for OPG, OPG ligand (OPGL), and osteoclast differentiation factor (ODF) were identified [9, 10]. It was later shown that both OPGL and ODF were identical to RANKL. The ODF receptor (ODFR) was shown to be a signaling receptor for ODF and identical to RANK [11]. The triad of the ligand/signaling receptor/decoy receptor is now called RANKL/RANK/OPG.

The studies above together with later studies revealed the pivotal roles of RANKL, RANK, and OPG in both bone metabolism and the immune system. In addition, these molecules have been shown to be involved in diverse physiological and pathological contexts.

## The structures of RANKL/RANK/OPG

RANKL, RANK, and OPG belong to the TNF and its receptor superfamilies. As a TNF superfamily molecule, RANKL forms a homotrimer and binds to its receptors. RANK and OPG act as a monomer and homodimer, respectively. The crystal structures of the RANK–RANKL and OPG–RANKL complex have been resolved at 2.7 Å resolution [12].

### RANKL

The human RANKL gene (gene symbol: *TNFSF11*) is located on chromosome 13 (13q14.11) and encodes a glycoprotein with 317 amino acids. Human and mouse RANKL share 85% identity in their amino acid sequences. RANKL belongs to the TNF cytokine superfamily. RANKL is a type II transmembrane protein with an extracellular domain at the carboxy–terminus [1, 2]. This ectodomain is cleaved by enzymes such as matrix metalloproteinases and released to the extracellular environment as soluble RANKL. Both membrane-bound and soluble RANKL bind to RANK, but the former seems to be more functionally significant than the latter at present (see below) [13–17].

### RANK

The human RANK gene (gene symbol: *TNFRSF11A*) is located on chromosome 18 (18q21.33) and encodes a receptor with 616 amino acids. Human and mouse RANK share 66% identity in their amino acid sequences. RANK belongs to the TNF receptor superfamily. The extracellular and intracellular domains of RANK contain four cysteine-rich pseudorepeats at the amino–terminus and three TRAF-binding domains at the carboxy–terminus, respectively [1, 2]. RANK is mainly expressed in osteoclast precursors, mature osteoclasts, and immune cells such as DCs, macrophages, and microglia. A recent study demonstrated that the osteoclast releases RANK-expressing extracellular vesicles, which interact with the RANKL on osteoblasts. The interaction results in the promotion of bone formation by RANK–RANKL reverse signaling [18].

### OPG

The human OPG gene (gene symbol: *TNFRSF11B*) is located on chromosome 8 (8q24.12) and encodes a receptor with 401 amino acids. Human and mouse OPG share 85% identity in their amino acid sequences. OPG also belongs to the TNF receptor superfamily. The domains of OPG contain four cysteine-rich pseudorepeats at the amino-terminus and two death domains at the carboxy-terminus, respectively [1, 2]. OPG is exported into the extracellular space as a soluble decoy receptor without any transmembrane structure.

## RANKL in bone metabolism

Bone undergoes a cycle of osteoclastic bone resorption and osteoblastic bone formation, i.e., the process of bone remodeling. The osteoclast is a large multinucleated cell that degrades the bone matrix with acid and catalytic enzymes. Osteoclasts are derived from monocyte/macrophage lineage cells by stimulation with the essential cytokine for osteoclastogenesis, RANKL [2, 19].

### Bone development

**Fig. 1:**
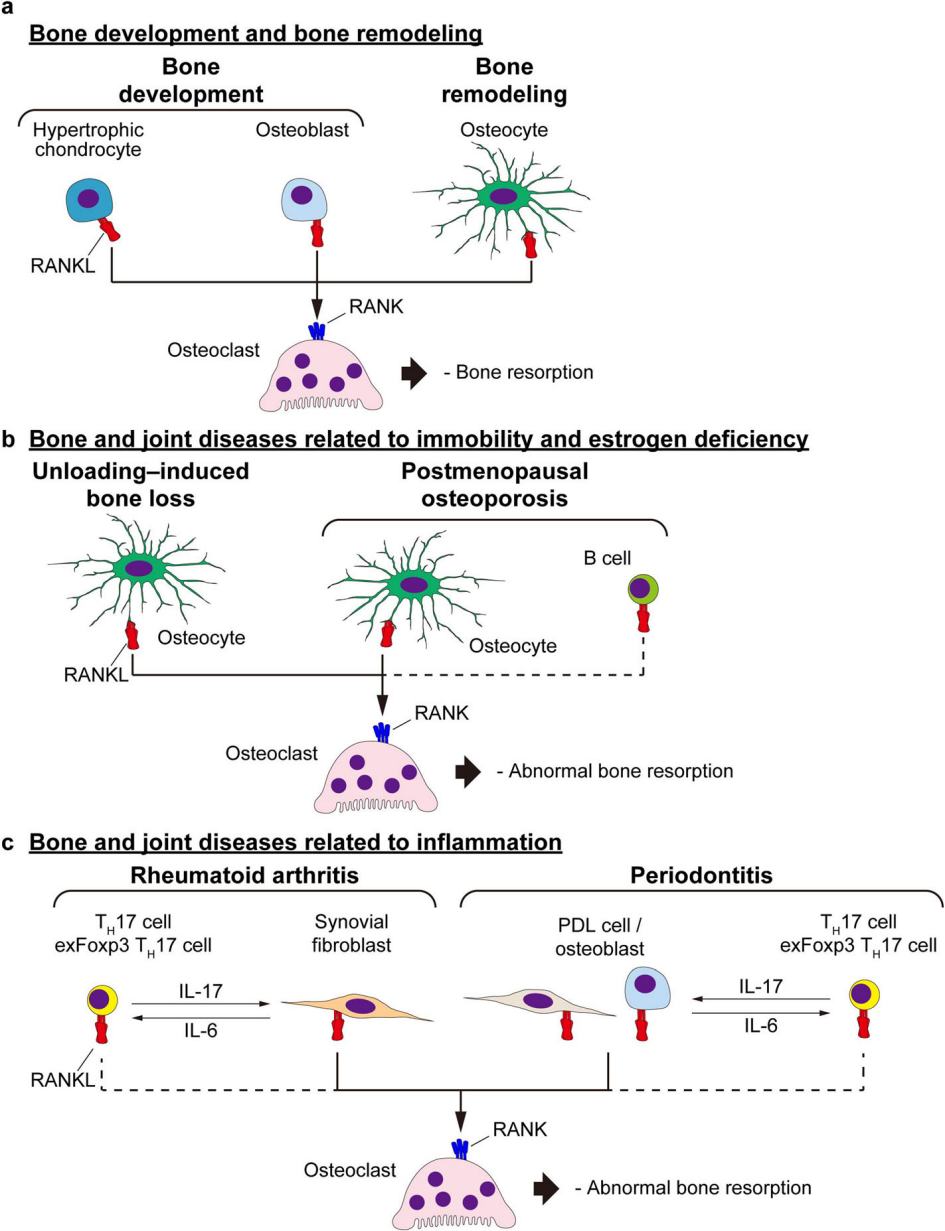
RANKL in bone metabolism. **a** The RANKL–RANK interaction in bone development and remodeling. Hypertrophic chondrocytes and osteoblasts function as the source of RANKL during growth. After the growth period, osteocytes are the major source of RANKL. RANKL induces the differentiation of osteoclasts, which resorb bone matrix. **b** RANKL–RANK interaction in bone and joint diseases related to immobility and aging. The bone loss induced by unloading is induced by osteocyte RANKL. B cell RANKL is reported to partially contribute to the bone loss in postmenopausal osteoporosis, as well. **c** In the lesion that occur in rheumatoid arthritis, synovial fibroblasts stimulated with pro-inflammatory cytokines, including IL-17, express RANKL and enhance osteoclastogenesis. In periodontitis, RANKL is mainly provided by PDL cells and osteoblasts. (see also Table 2). The IL-17 in these processes is produced by T_H_17 cells stimulated by IL-6. T_H_17 cells (exFoxp3 T_H_17 cells, in particular) express RANKL as well. *RANKL* receptor activator of NF-κB ligand, *RANK* receptor activator of NF-κB, *T*_*H*_*17 cell* T helper 17 cell, *PDL* periodontal ligament

In bone tissue, RANKL is expressed by several types of cells including osteoblasts, osteocytes and immune cells. Among these cells, RANKL expression is higher in osteoblasts and osteocytes. In neonatal or young mice in their growth period, hypertrophic chondrocytes in the growth plate and osteoblasts are the major sources of RANKL. In older mice, on the other hand, osteocytes contribute more to RANKL expression (Fig. 1a) [20–22]. RANKL binds to its corresponding receptor RANK, thereby inducing subsequent osteoclastogenic signals.

### Hereditary bone diseases

Because of its essentiality in osteoclastogenesis, dysregulation of RANKL signaling results in excessive or impaired bone resorption, and certain therapeutic interventions in such dysregulated signaling have been shown to be effective in the treatment of bone diseases [1]. Mutations in genes encoding RANKL, RANK, and OPG lead to hereditary bone diseases in human, such as autosomal recessive osteopetrosis (ARO) [23, 24], familial form of early-onset Paget’s disease of bone (PDB2) [25–27], familial expansile osteolysis (FEO) [26, 28–30], expansile skeletal hyperphosphatasia (ESH) [31], panostotic expansile bone disease (PEBD) [32], and the Juvenile Paget’s disease (JPD, or idiopathic hyperphosphatasia, IH) [32–37]. Mutations found in these diseases are summarized in Table 1.

**Table 1:** Mutations of RANKL/RANK/OPG genes in hereditary bone diseases

Molecule	Gene symbol	Location	Site of mutation		Disease	Literature
			DNA	Protein		
RANKL	*TNFSF11*	13q14	IVS7+4_8del		Osteopetrosis, autosomal recessive 2	23
			c.596T>A	p.Met199Lys	Osteopetrosis, autosomal recessive 2	23
			c.828_829delCG	p.Val277fs	Osteopetrosis, autosomal recessive 2	23
RANK	*TNFRSF11A*	18q21.33	c.40_66dup	p.Ala13_Leu21dup	Paget disease of bone 2, early–onset	25, 26
			c.48_65dup	p.Leu16_Leu21dup	Familial expansile osteolysis	28, 29, 30
			c.49_63dup	p.Leu16_Leu20dup	Expansile skeletal hyperphosphatasia	31
			c.49_66dup	p.Leu16_Leu21dup	Familial expansile osteolysis	26
			c.52_66dup	p.Leu16_Leu21dup	Paget disease of bone 2, early–onset	27
			c.55_66dup	p.Cys18_Leu21dup	Panostotic expansile bone disease	32
			c.157G>C	p.Gly53Arg	Osteopetrosis, autosomal recessive 7	24
			c.385C>T	p.Arg129Cys	Osteopetrosis, autosomal recessive 7	24
			c.508A>G	p.Arg170Gly	Osteopetrosis, autosomal recessive 7	24
			c.523T>C	p.Cys175Arg	Osteopetrosis, autosomal recessive 7	24
			c.730G>T	p.Ala244Ser	Osteopetrosis, autosomal recessive 7	24
OPG	*TNFRSF11B*	8q24.12	100 kb deletion		Paget disease of bone 5, juvenile–onset	33
			245 kb deletion		Paget disease of bone 5, juvenile–onset	37
			c.193T>C	p.Cys65Arg	Paget disease of bone 5, juvenile–onset	35
			c.226A>C	p.Thr76Pro	Paget disease of bone 5, juvenile–onset	37
			c.260G>A	p.Cys87Tyr	Paget disease of bone 5, juvenile–onset	35
			c.349T>C	p.Phe117Leu	Paget disease of bone 5, juvenile–onset	35
			c.592_IVS3+19_20del		Paget disease of bone 5, juvenile–onset	35
			c.544_546delGAC	p.Asp182del	Paget disease of bone 5, juvenile–onset	34, 35
			c.966_969delTGAC insCTT	p.Asp323Ser fsX3	Paget disease of bone 5, juvenile–onset	36

*IVS* intervening sequence, *c*. coding DNA, *p*. protein, *del* deletion, *dup* duplication, *ins* insertion, *fs* frame shift

### Bone remodeling under the influence of mechanical loading

Mechanical loading onto bone maintains its morphology, quantity, and quality. In cases of being bed-ridden or undergoing spaceflight, the body endures reduced mechanical loading, resulting in increased osteoclastic bone resorption and fragility. It is reported that unloading-induced osteoclastic bone resorption is mediated by osteocyte RANKL (Fig. 1b) [21]. On the other hand, bone remodeling by additional mechanical loading has been used in orthodontic treatment for a long time. Orthodontic force applied to teeth induces alveolar bone remodeling so that the selected teeth move toward the targeted destination. During such alveolar bone remodeling, osteocytes function as the major source of RANKL [38]. Thus, as described above, both unloading and loading conditions can induce the osteoclastic bone resorption, which is mediated by the increase of osteocyte RANKL. The mechanism of precisely how this cytokine is induced in osteocytes requires further study.

### Osteoporosis

Osteoporosis is defined as a disease characterized by low bone mass and microarchitectural deterioration of bone tissue caused by an unbalancing of the resorption-formation toward resorption [39]. This imbalance is induced by alterations in hormone expression, nutrition, mobility, and/or senescence. Diseases and medication used to treat them can result in osteoporosis as well. Studies have shown that B cell RANKL, as well as osteocyte RANKL, to some extent contributed to bone loss in a mouse model of postmenopausal osteoporosis, whereas that of T cells did not (Fig. 1b) [40, 41]. Recently, it was reported that soluble RANKL deficiency did not affect the severity of bone loss in this model, suggesting a role for membrane-bound RANKL to the pathology of osteoporosis [16, 17]. Because inhibition of RANKL can ameliorate excessive bone resorption by suppressing osteoclastogenesis, a human monoclonal IgG2 antibody against RANKL denosumab has come to be used for the treatment of osteoporosis over the last decade in many countries [42, 43]. Romosozumab, a monoclonal antibody against sclerostin, has started to be used for osteoporosis patients very recently [44]. Sclerostin is a well-known inhibitor of Wnt signaling, and its neutralization leads to an increased bone formation. In addition, sclerostin was shown to induce RANKL expression [45, 46], and romosozumab decrease bone resorption via its inhibition.

### Inflammatory bone loss

Rheumatoid arthritis (RA) is a joint disease characterized by chronic inflammation of the synovium and erosion of cartilage and bone [47]. In this context, RANKL that mediate osteoclastogenesis is produced by the synovial fibroblasts under inflammation, as well as T helper 17 (T_H_17) cells, especially those that with a history of Foxp3 expression (exFoxp3 T_H_17 cells) (Fig. 1c) [48–50]. Denosumab has been shown to be effective in inhibiting the progression of joint destruction [51], but its clinical use is approved in only a limited number of countries. Because denosumab was effective in the prevention of bone destruction but not joint inflammation or cartilage destruction, it is desirable to use this drug in combination with others, such as methotrexate and biologics [52].

Periodontitis is the most common infectious disease and the major cause of tooth loss owing to the loss of tooth-supporting bone, alveolar bone [53]. Bacterial penetration of the oral epithelium leads to an immune response in the periodontium, generating exFoxp3 T_H_17 cells [15]. These cells produce interleukin (IL)-17 to stimulate osteoblasts and periodontal ligament (PDL) cells to express RANKL, as well as other inflammatory cytokines, resulting in osteoclast generation and subsequent bone destruction (Fig. 1c). The bone destruction similarly occurs in mice deficient in soluble RANKL [15]. The loss of alveolar bone eventually leads to ejection of the teeth and resultant alleviation of inflammation [54]. Sources of RANKL in these contexts are summarized in Table 2.

**Table 2:** Table caption

Context	Model	Source		Contribution	Reference
Disuse atrophy	Tail suspension (mouse)	Osteocyte	(*Dmp1*–expressing)	+	21
Orthodontic tooth movement	Orthodontic tooth movement (mouse)	Osteocyte	(*Dmp1*–expressing)	+	38
Osteoporosis	Ovariectomy (mouse)	B cell	(*Cd19*–expressing)	Partial	40
Osteoporosis	Ovariectomy (mouse)	T cell	(*Lck*–expressing)	–	40
Osteoporosis	Ovariectomy (mouse)	Osteocyte	(*Dmp1*–expressing)	+	41
Rheumatoid arthritis	Collagen antibody-induced arthritis (mouse)	Synovial fibroblast	(*Col6a1*–expressing)	+	48
Rheumatoid arthritis	Collagen antibody-induced arthritis (mouse)	T cell	(*Lck*–expressing)	–	48
Rheumatoid arthritis	Collagen antibody-induced arthritis (mouse)	Articular chondrocyte	(*Col2a1*–expressing)	–	48
Rheumatoid arthritis	Collagen-induced arthritis (mouse)	Synovial fibroblast	(*Col6a1*–expressing)	+	48
Rheumatoid arthritis	Collagen-induced arthritis (mouse)	T cell	(*Lck*–expressing)	–	48
Periodontitis	Ligature–induced periodontitis (mouse)	B cell	(*Mb1*–expressing)	–	15
Periodontitis	Ligature–induced periodontitis (mouse)	T cell	(*Cd4*–expressing)	Partial	15
Periodontitis	Ligature–induced periodontitis (mouse)	Periodontal ligament cell	*(Scx–expressing)*	+	15
Periodontitis	Ligature–induced periodontitis (mouse)	Osteoblast–lineage cell	*(Sp7–expressing)*	+	15

As described above, the RANKL–RANK system plays a crucial role in bone resorption, dysregulation, and re-regulation of which are therefore the key element in both bone diseases and their treatments. Recently, the vesicular RANK secreted from osteoclasts was revealed to promote osteoblastogenesis by activating Runx2 via RANK–RANKL reverse signaling [18]. With this finding, the RANKL–RANK system attained greater significance for bone biology.

## RANKL in immunity

RANKL signaling is crucial for the development of various organs, including immune organs. In fact, RANKL was first reported as an activator of dendritic cells expressed by T cells [4]. The immune organs consist of immune cells and stromal cells. Studies using mice have shown that several of these cell types express RANKL or RANK, transducing signals for the development and function of the immune system as described below.

### Bone marrow formation

The bone marrow is one of the primary lymphoid organs, where lymphocytes emerge and mature. Both T and B cells are born in the bone marrow and the latter cells mature in this organ. Other types of hematopoietic cells including erythrocytes reside in this space as well. Because the bone marrow space is preserved by osteoclastic bone resorption within the bone, RANKL functions as a maintainer of the bone marrow and its indwelling immune cells. In most types of osteopetrosis, the patients exhibit mild to severe hematological defects, which can lead to anemia, hemorrhage, and severe or recurrent infectious diseases [55, 56].

### Thymus development

**Fig. 2:**
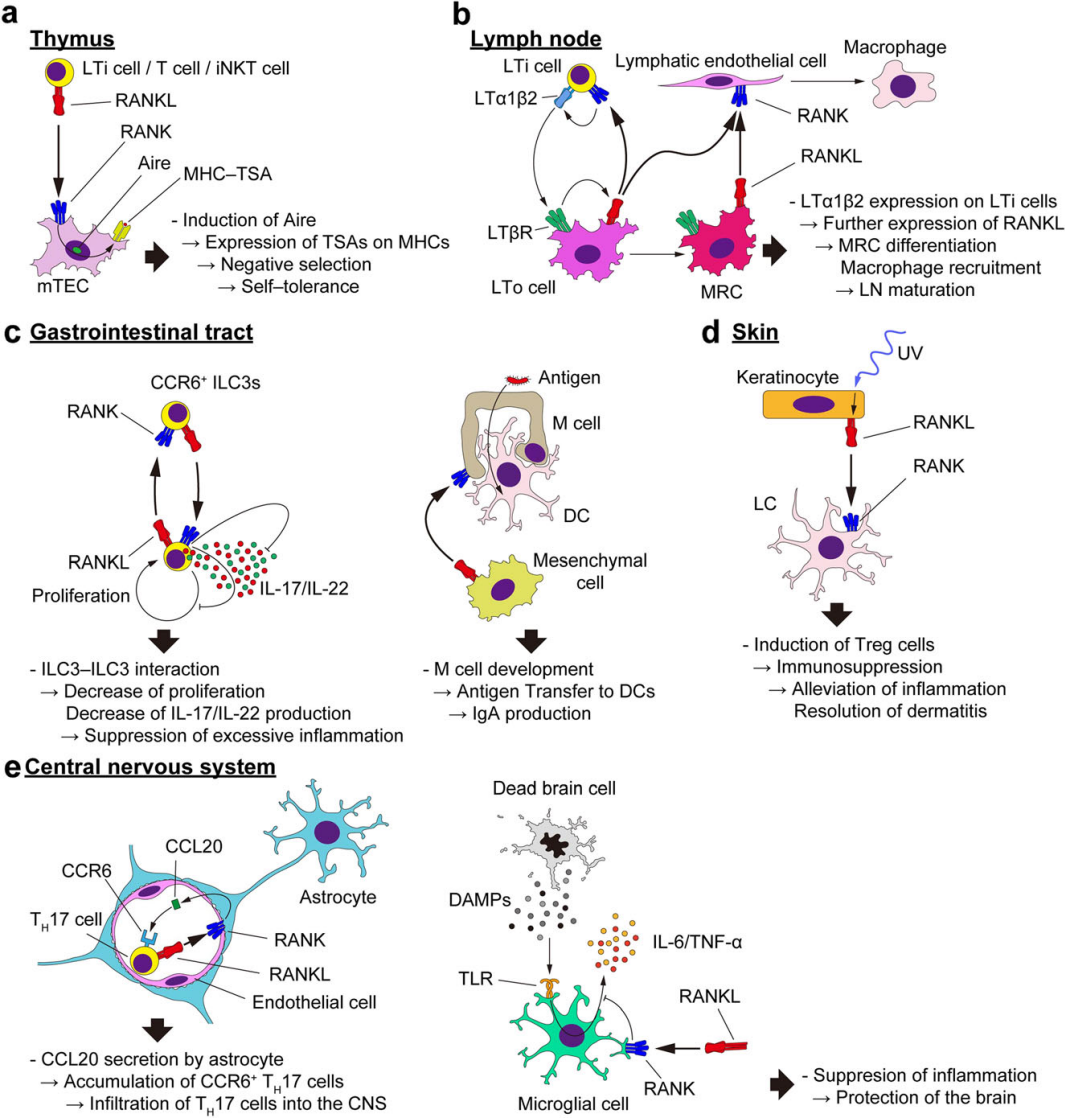
RANKL in immunity. **a** RANKL–RANK interaction in the development of the thymus. RANKL is produced by LTi cells, T cells, and iNKT cells and interacts with the RANK expressed on mTECs. This interaction induces the expression of Aire, resulting in the expression of TSAs on MHC molecules. The TSA–MHC complex is necessary for negative selection, the key process for establishing self-tolerance. **b** RANKL–RANK interaction in the lymph node development. Lymph node development begins with the interaction between LTi cells and LTo cells. LTα1β2 is expressed by LTi cells and interacts with LTβR on LTo cells, which in turn leads to the expression of RANKL on LTo cells. The expressed RANKL stimulates LTi cells to induce more LTα1β2, forming a positive feedback loop. With the stimulation of LTα1β2, some LTo cells mature into MRCs. The RANKL on LTo cells and MRCs binds to the RANK on lymphatic endothelial cells, resulting in the recruitment of macrophages. **c** RANKL–RANK interaction in the gastrointestinal tract. (Left) ILC3s interact with each other through RANKL and RANK. The interaction leads to the decrease of the proliferation and IL-17/IL-22 production of these cells, resulting in the suppression of excessive inflammation. (Right) RANKL–RANK interaction in M cell development. Mesenchymal cells beneath the epithelium of the gastrointestinal tract express RANKL and interact with RANK–expressing epithelial cells. These cells differentiate into morphologically and functionally unique cells called M cells. These cells enable the transfer of antigens from the lumen of gastrointestinal tract to DCs, leading to IgA production. **d** RANKL–RANK interaction in the skin. Keratinocytes express RANKL upon UV–irradiation. The RANKL binds to LCs in the skin. These LCs contribute to the generation of Treg cells, which decrease the skin inflammation and resolution of dermatitis in psoriasis and atopic dermatitis. **e** RANKL–RANK interaction in the CNS inflammation. (Left) T_H_17 cell cells induce the CCL20 expression of astrocytes at the blood–brain barrier via RANKL–RANK signaling. CCL20 recruits CCR6-expressing cells, including T_H_17 cell cells. These accumulated cells penetrate the barrier and infiltrate into the CNS to elicit inflammation. (Right) In the context of ischemic stroke, dead cells in the brain release DAMPs, which are recognized by TLRs. TLR stimulation of microglial cells leads to the production of pro-inflammatory cytokines including IL-6 and TNF-α, leading to inflammation and further cell death. RANKL–RANK signal in the microglial cells inhibits the production of these cytokines, resulting in the protection of the brain. *RANKL* receptor activator of NF-κB ligand, *RANK* receptor activator of NF-κB, *LTi cell* lymphoid tissue inducer cell, *iNKT cell* invariant natural killer T cell, *mTEC* medullary thymic epithelial cell, *Aire* autoimmune regulator, *TSA* tissue-specific antigen, *MHC* major histocompatibility complex, *LTo cell* lymphoid tissue organizer cell, *LT* lymphotoxin, *LTβR* lymphotoxin β receptor, *MRC* marginal reticular cell, *ILC3* group 3 innate lymphoid cell, *IL* interleukin, *DC* dendritic cell, *UV* ultra violet, *LC* Langerhans cell, *Treg cell* regulatory T cell, *CNS* central nervous system, *T*_*H*_*17 cell* T helper 17 cell, *CCL20* C-C motif chemokine ligand 20, *CCR6* C-C motif chemokine receptor 6, *DAMP* damage-associated molecular pattern, *TLR* Toll-like receptor

The thymus is another primary lymphoid organ where T cell progenitors undergo the positive and the negative selections for acquiring the property to distinguish non-self from self-antigens, thereby establishing self-tolerance. During negative selection, cells that strongly interact with the self-antigens expressed on major histocompatibility complex (MHC) molecules undergo apoptosis [57]. In this process, these antigens, including a portion of the tissue-specific antigens (TSAs), are expressed by medullary thymic epithelial cells (mTECs) under the control of a crucial factor, autoimmune regulator (Aire) [58, 59]. RANKL is a key cytokine for inducing Aire expression in these epithelial cells, and it is provided by lymphoid tissue inducer (LTi) cells, single positive thymocytes, Vγ5^+^ γδ T cells, and invariant natural killer T (iNKT) cells (Fig. 2a) [60–63]. Because thymic development is normal in mice deficient in soluble RANKL, it is suggested that membrane-bound RANKL in these cells induces mTEC development [17].

### Lymph node development

RANKL also contributes to the development and function of the secondary lymphoid organs, where immune responses take place. The LN is one such organ distributed throughout the body. LNs consist of lymphocytes and their surrounding stromal cells, establishing a complex but well-organized structure, with B and T cells localized in distinct regions [64]. LN organogenesis begins with the condensation of LTi cells, which are CD45^+^CD4^+^CD3^−^IL-7R^+^RORγt^+^, and specific mesenchymal cells named lymphoid tissue organizer (LTo) cells. RANKL is expressed on LTi cells, LTo cells, and the descendants of the latter, marginal reticular cells (MRCs) [65, 66]. The expression of RANKL on the stromal cells in the LNs is reported to be enhanced by lymphotoxin β receptor (LTβR) signaling [67]. The RANKL signal, more likely via the membrane-bound type [17], induces the maturation of the LNs by increasing cellularity and the attraction of immune cells to the LNs [6, 65]. It was recently reported that the RANKL expressed by LTo lineage cells stimulate lymphatic endothelial cells to recruit and maintain macrophages in the LNs (Fig. 2b) [68].

### Intestinal immunity

The gastrointestinal (GI) tract is the largest pathogenic bacteria entry site, with a surface area 100 times that of the body surface. In order to protect the body from these bacteria, the GI tract has developed a highly specialized defense system. Lymphocytes lacking antigen receptors, innate lymphoid cells (ILCs), are known to be abundant in the mucosal tissues and constitute a part of barrier functions by secreting cytokines [69, 70]. Group 3 ILCs, including LTi cells and ILC3s, express a transcription factor RORγt and produce high amount of cytokines IL-17 and IL-22, contributing to the homeostasis in the intestine [71, 72]. A recent study reported that ILC3s are divided into NKp46^−^CCR6^−^, NKp46^+^CCR6^−^, and NKp46^−^CCR6^+^ cells. The expression of both RANKL and RANK showed the highest in the CCR6^+^ cells, which cluster within the cryptopathces [73, 74]. The proliferation and IL-17A/IL-22 expression of the CCR6^+^ ILC3s were suppressed by RANKL [73], indicating that these cells interact with each other in the cryptopatches to suppress excessive proliferation and inflammation (Fig. 2c).

Peyer’s patches (PPs) are lymphoid follicles beneath the intestinal epithelium. Within the epithelium covering the PPs (follicle-associated epithelium, FAE), there is a unique cell subset, M cells. Unlike their surrounding epithelial cells, M cells lack villi, but have a micro-fold structure on the apical side and a sac-like structure (the M-cell pocket) on the basal side. These cells have a high capacity for transcytosis, thus transferring the bacteria in the lumen to the DCs in the M-cell pocket. Antigen presentation to DCs via M cells results in the immune response to the transcytosed bacteria, i.e., IgA production [75]. RANKL is necessary and sufficient for M cell development, and its source during the process has been shown to be the mesenchymal cells in the lamina propria (Fig. 2c). The deficiency in soluble RANKL has not affected the development of these cells [76]. The RANKL in these mesenchymal cells also plays a role in IgA production [14].

### Skin inflammation

The skin is the front line of the defense against external stimuli, and is thus equipped with a specific immune system. Langerhans cells (LCs) reside in the epidermis and are one of the key components of skin immunity [77, 78]. LCs are classified as a DC subset, with neuron-like dendrites, a high capacity for antigen presentation, and a capacity to migrate into the LNs, where LCs present antigens to T cells, thereby generating inflammatory or regulatory T (Treg) cells. RANKL has been shown to be expressed by keratinocytes upon ultra violet (UV) irradiation via the prostaglandin E receptor (EP) 4 signal [79]. The RANKL expressed by the keratinocytes interacts with RANK on LCs, resulting in the expansion of Treg cells. The increased Treg cells exert immunosuppressive effects [80], decreasing excessive inflammation in the skin (Fig. 2d). The immunosuppression induced by UV is the basis of the phototherapy used for psoriasis and atopic dermatitis, but is also can lead to carcinogenesis [81].

### Inflammation in the central nervous system

The central nervous system is an immune-privileged site, which is due to the presence of the blood–brain barrier (BBB) comprised of endothelial cells, pericytes, and astrocytes. This barrier restricts the entry of cells and microorganisms [82]. A study showed that penetration of the BBB by pathogenic T_H_17 cells in a multiple sclerosis mouse model depended on RANKL signaling; T_H_17 cells expressing RANKL interact with RANK-expressing astrocytes, which in turn secrete C-C motif chemokine ligand 20 (CCL20), further attracting C-C motif chemokine receptor 6 (CCR6)-expressing cells into the central nervous system (CNS) (Fig. 2e) [83].

In the brain tissue with ischemic stroke, there is an inflammation elicited by immune cells including microglial cells, macrophages, DCs, and γδ T cells [84, 85]. Reduced blood flow in the brain leads to the brain cell death, which results in the release of damage-associated molecular patterns (DAMPs) form the dead cells. These DAMPs include high mobility group box-1 (HMGB1) and peroxiredoxin (Prx), which lead to the BBB break and the stimulation of the immune cells above [86]. Clinical studies have observed that serum OPG concentration is higher in patients with ischemic stroke and is positively correlated with the severity [87]. A study showed that RANKL suppresses the production of pro-inflammatory cytokine, such as IL-6 and TNF-α, induced via Toll-like receptor 4 (TLR-4) (Fig. 2e) [84].

The course of these studies has revealed that the RANKL signal functions in various immune settings such as organogenesis, immune cell development, as well as the regulation of their function. Because RANKL serves sometimes beneficial but other times harmful, the modulation of this cytokine may be therapeutic utility in diseases affecting the immune system. Careful studies are needed to avoid the potential occurrence of side effects.

## RANKL involvement in other biological processes

It has become clear that the RANKL/RANK system not only plays an important role in bone metabolism and the immune system, but it also has various physiological functions in multiple other organs.

### Mammary gland development and function

**Fig. 3:**
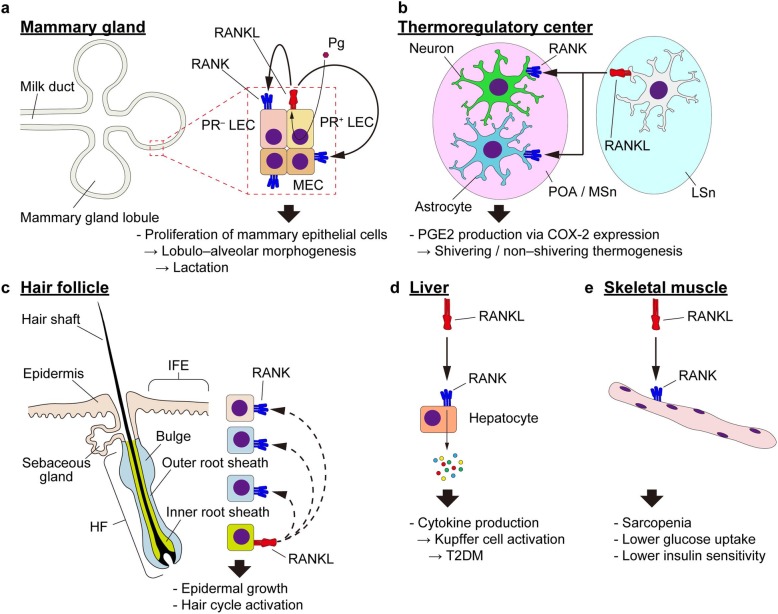
RANKL in biological processes other than bone metabolism and the immune systems. **a** RANKL–RANK interaction in the development of the mammary gland. The LECs of the mammary gland are divided into two subpopulations based on the expression of PR. PR-expressing LECs express RANKL in response to Pg. RANKL interacts with LECs and MECs, resulting in the proliferation of these epithelial cells and the morphogenesis of the gland. **b** RANKL–RANK interaction in thermogenesis. Certain types of cells of the LSn of the forebrain express RANKL, which interacts with neurons and astrocytes in the POA and the MSn. These nuclei produce PGE2 via COX-2, which leads to both shivering and non-shivering thermogenesis. **c** RANKL–RANK signaling in the blood vessel. Both RANKL and RANK are expressed on vascular cells including VSMCs. RANKL induces the expression of BMP2 and 4, which promotes the osteogenic gene expression of these cells, resulting in the vascular calcification. The signal is suppressed by estrogen and its receptor ERα. Expression of RANKL and RANK in this context is enhanced by Ang II. Production of Ang II is increased in turn by RANKL and RANK. **d** RANKL–RANK interaction in the hair cycle. Cells in the inner root sheath of the HF express RANKL. Cells in the outer root sheath, the bulge and the IFE express RANK. The interaction of these cells induces the growth of the epidermis and activates the hair cycle. **e** RANKL–RANK interaction in the liver. Hepatocytes stimulated with RANKL express pro-inflammatory cytokines that stimulate Kupffer cells, leading to T2DM. **f** RANKL–RANK interaction in the skeletal muscle. RANKL–RANK signaling in muscle fibers is involved in the strength and glucose metabolism of the skeletal muscle. *RANKL* receptor activator of NF-κB ligand, *RANK* receptor activator of NF-κB, *Pg* progesterone, *PR* progesterone receptor, *LEC* luminal epithelial cell, *MEC* myoepithelial cell, *LSn* lateral septal nucleus, *POA* preoptic area, *MSn* medial septal nucleus, *PGE2* prostaglandin E2, *COX*-2 cyclooxygenase-2, *VSMC* vascular smooth muscle cell, *BMP* bone morphogenetic protein, *ER* estrogen receptor, *Ang* angiotensin, *ATR* angiotensin receptor, *HF* hair follicle, *IFE* interfollicular epidermis, *T2DM* type 2 diabetes mellitus

The mammary gland undergoes morphological changes in pregnancy to allow lactation. During pregnancy, there is extensive lateral branching and epithelial bud development of epithelial buds, which are organized into secretory lobular structures in preparation for lactation. From an analysis of RANKL- or RANK-deficient mice, it was revealed that the RANKL/RANK system is also important for the formation of the lactating mammary gland [88]. Although the mammary gland develops normally in RANKL-deficient mice, the formation of lobuloalveoli, which are capable of milk secretion during pregnancy, was blocked due to a suppression of the proliferation of the mammary epithelium. Mechanistically, RANKL promotes the proliferation of mammary epithelial cells through the expression cyclin D1 by activating NF-κB [89]. In addition, progesterone, which is an essential sex hormone for the proliferation of adult mammary epithelial cells and the formation of milk-secreting acini, directly regulates RANKL expression in mammary epithelial cells through the progesterone receptor, and the RANKL secreted from these cells binds to RANK in both an autocrine and paracrine manner [90]. The RANKL/RANK system has also been shown to control mammary stem cell (MaSC) replication. Although the progesterone receptor is not expressed in MaSCs, progesterone acts on its receptor expressed in luminal epithelial cells to induce RANKL expression, which increases the pool of MaSCs by acting on RANK-expressing basal epithelial cells in a paracrine manner (Fig. 3a) [90].

### Fever and the regulation of body temperature

Both RANKL and RANK are expressed in the central nervous system, but their function was for a long time unknown. RANK is specifically expressed in neurons and astrocytes in the preoptic area (POA)/medial septal nucleus (MSn), whereas RANKL is expressed in the lateral septal nucleus (LSn) [91]. These sites were known to be involved in central control of fever and body temperature. Indeed, stereotactic intracerebroventricular injections of recombinant RANKL into the lateral ventricle of mice led to a febrile reaction. Since this effect was canceled by either treatment with indomethacin, a non-selective cyclooxygenase (COX)-1/2 inhibitor, or a genetic deletion of EP3, a receptor for prostaglandin E2 (PGE2), the thermoregulatory mechanism of the RANKL/RANK axis in the brain is mediated by central prostaglandin synthesis. Moreover, RANK deficiency abolished the LPS-induced fever, suggesting that the central RANKL/RANK signaling also mediates the inflammatory fever response (Fig. 3b). It was also shown that the RANKL/RANK-mediated control of thermoregulation is involved not only in the fever that occurs during infection but also in the hormonal control of basal body temperature in females.

### Vascular calcification

There is growing evidence that the RANKL/RANK/OPG system is related to vascular calcification. The expression of RANKL/RANK/OPG is upregulated in calcified arteries and that RANKL promotes pathological differentiation of vascular smooth muscle cells (VSMCs) into cells with osteoblastic phenotype, at least in part, through the expression of bone morphogenetic protein (BMP) 4 [92]. The administration of OPG decreased calcification and the expression of osteogenic genes in aortic valves in a mouse model of atherosclerosis. Moreover, the effects of RANKL on vascular cells are suppressed by estrogen signaling. In ovariectomized ApoE-deficient mice, estrogen treatment inhibited vascular calcification as a result of the inhibition of BMP/Smad signaling [93]. It was also reported that RANKL expression and calcification in VSMCs were increased by angiotensin II. Since vascular calcification was suppressed by the administration of an angiotensin II receptor antagonist, the local renin-angiotensin system contributes to vascular calcification through the expression of RANKL. Conversely, stimulation of VSMCs with RANKL increased the expression of angiotensin II receptor and angiotensin-converting enzyme [94]. These results suggest that the RANKL/RANK/OPG system may contribute to the formation of vascular calcification at the site of atherosclerosis (Fig. 3c)

### Hair growth

The RANKL/RANK system also plays an important role in hair follicle development in mice [95]. Although RANKL and RANK are expressed in the interfollicular epidermis (IFE) and hair follicles (HFs) of the epidermo–pilosebaceous unit during development, RANKL/RANK signaling is in fact dispensable for HF morphogenesis. On the other hand, the HFs in RANK- or RANKL-deficient mice are unable to initiate the anagen (growth) phase of the hair regeneration cycle. Transgenic expression of RANK in the HFs or subcutaneous injection of recombinant RANKL activates the hair cycle and epidermal growth. RANKL is highly expressed in HFs at the initiation of the anagen phase and drives the HF stem cells into proliferation (Fig. 3d).

### Glucose metabolism

It has been shown that the RANKL/RANK system is also related to the pathogenesis of type 2 diabetes mellitus (T2DM). The serum level of soluble RANKL was shown to be a significant risk predictor of T2DM in a large prospective study [96]. Blockage of RANKL or RANK either systemically or specifically in the liver of T2DM mouse models leads to a significant improvement of hepatic insulin sensitivity, plasma glucose concentrations, and glucose tolerance. RANKL/RANK signaling activates NF-κB in hepatocytes, leading to inflammatory cytokine production, Kupffer cell activation, and excess storage of fat (Fig. 3e).

### Muscle strength

RANK is also known to be expressed in skeletal muscle. The activation of RANKL/RANK signaling in skeletal muscle leads to the inhibition of myogenic differentiation through the activation of NF-κB, which results in skeletal muscle dysfunction and loss [97]. In fact, administration of the recombinant OPG protein improved muscle strength in a mouse model of Duchenne’s muscular dystrophy and denervation-induced muscle atrophy. More recently, the effect of RANKL/RANK inhibition on muscle mass and strength was also reported, particularly in conditions of osteoporosis or sarcopenia [98]. Mice carrying the human RANKL genomic region (huRANKL–Tg mice) displayed decreased muscle mass, force, fat infiltration, and glucose uptake, along with low bone mass phenotype and upregulation of antimyogenic and inflammatory genes. The administration of the recombinant OPG protein or denosumab restored muscle mass, function, and glucose utilization in huRANKL–Tg mice as well as peroxisome proliferator-activated receptor β (PPARβ)-deficient mice, which develop a combination of sarcopenia and a low bone mass phenotype. It was also shown that denosumab treatment for more than 3 years improved the appendicular lean mass and handgrip strength of osteoporotic women. Thus, RANKL/RANK signaling decreases muscle strength, while denosumab treatment may preserve both bone and skeletal muscle function (Fig. 3f).

## RANKL in tumorigenesis and metastasis

It has become clear that the RANKL/RANK signaling is involved in a wide range of functions in the body. Furthermore, numerous studies have demonstrated RANKL and RANK expression in neoplastic tissues. Interestingly, the expression level of RANKL/RANK in cancer tissues is related to the prognosis of numerous cancer types, including breast, lung, endometrial, renal cell, and gastric cancer [99]. Therefore, the RANKL/RANK axis may influence the development and progression of cancer, while the specific effects of RANKL/RANK may differ between cancer types.

### Breast cancer

**Fig. 4:**
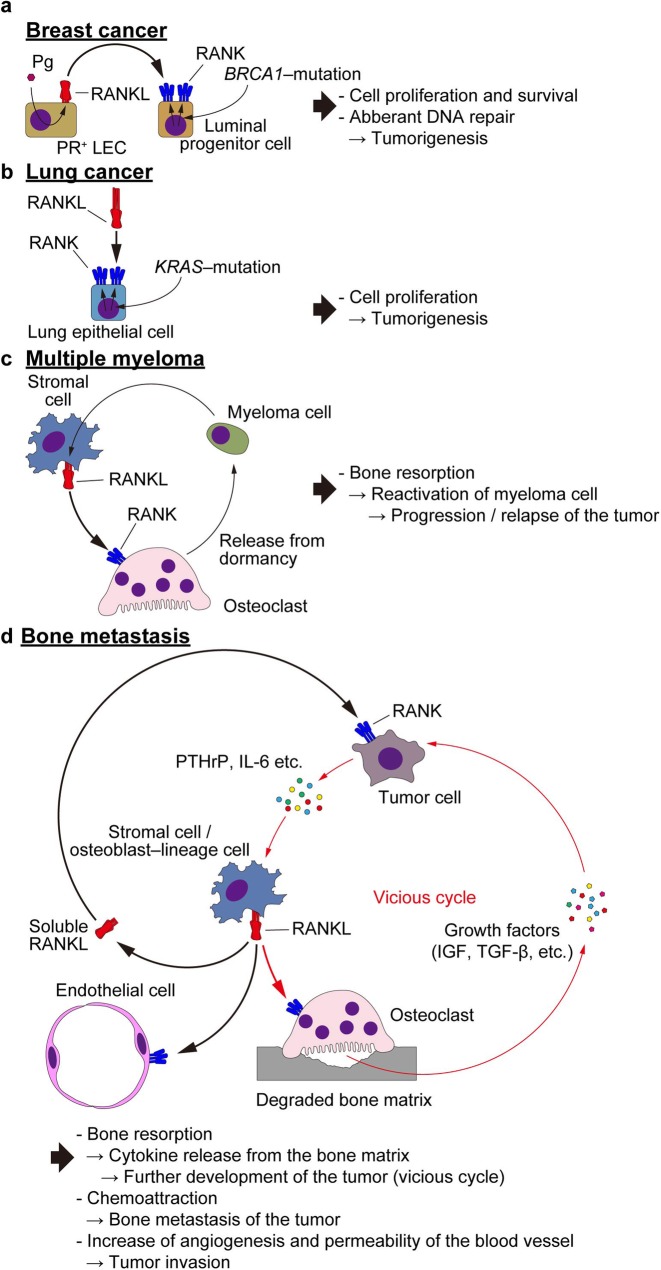
RANKL in tumorigenesis and metastasis. **a** RANKL–RANK interaction in breast cancer. Mutations in *BRCA1* lead to the increased expression of RANK in luminal progenitor cells of the mammary gland. The RANKL expressed on PR-expressing LECs (see Fig. 3a) stimulates the proliferation and survival of the mutant cells and DNA repair is impaired in these cells, resulting in the tumorigenesis. **b** RANKL–RANK interaction in lung cancer. *KRAS* mutations in the lung epithelial cells increase RANK expression on these cells. These cells undergo excessive proliferation upon RANKL stimulation, leading to tumor development. **c** RANKL–RANK interaction in multiple myeloma. Myeloma cells enhance RANKL expression on the stromal cells of tumors in the bone, resulting in osteoclastic bone resorption and the release of myeloma cells from dormancy. Together, these processes lead to an expansion of the tumors in the bone. **d** RANKL–RANK interaction in bone metastasis. Cancer cells metastasized to the bone marrow produce molecules, including PTHrP. Some of these induce RANKL expression on the tumor stromal cells. This RANKL induces osteoclastic bone resorption, and the degraded bone releases growth factors embedded in the matrix, such as IGF-1 and TGF-β. These factors increase the tumor size and the enlarged tumor further contributes to the amount of RANKL expression, forming a vicious cycle. The soluble form of RANKL contributes to the chemotaxis of the tumor cells expressing RANK toward the site of metastasis. Tumoral RANKL is also involved in the angiogenesis and the permeability of the blood vessels, facilitating tumor invasion. *RANKL* receptor activator of NF-κB ligand, *RANK* receptor activator of NF-κB, *Pg* progesterone, *PR* progesterone receptor, *LEC* luminal epithelial cell, *PTHrP* parathyroid hormone-related peptide, *IGF* insulin-like growth factor, *TGF*-*β* transforming growth factor-β

Breast cancer is the most common cancer in women. There is evidence that hormone replacement therapy is associated with an increased risk of breast cancer. Preclinical evidence suggests that RANKL/RANK signaling is involved in the oncogenic role of progesterone in the mammary gland [100, 101]. The drugs used as hormone replacement therapy or contraceptives induces RANKL expression in mammary epithelial cells, thereby increasing the proliferation of these cells and MaSCs. RANK overexpression under the control of the mouse mammary tumor virus (MMTV) promoter increased tumorigenesis of breast tissue induced by carcinogens or progesterone [100]. Consistent with this, RANKL inhibition by RANK–Fc led to a selective reduction in the proliferation of mammary epithelial cells and preneoplastic hyperplasia [100]. In mice with breast tissue-specific deletion of RANK, the tumorigenesis, tumor growth, and stem cell expansion driven by progestin were attenuated [101]. Furthermore, the RANKL/RANK signaling in mammary progenitor cells is critical for the initiation and progression of breast cancer susceptibility gene 1 (BRCA1) mutation-driven mammary cancer (Fig. 4a) [102, 103]. Targeting the RANKL/RANKL axis may be a rational prevention strategy for patients with BRCA1 mutation-positive breast cancer.

### Lung cancer

The RANKL/RANK signaling is also involved in lung cancer [104]. RANK and RANKL expression is frequently found in cells derived from lung cancer patients and have been associated with poor prognosis. Activation of the RANKL/RANK pathway regulates lung cancer stem-like cell expansion via a mechanism dependent on mitochondrial respiration (Fig. 4b). RANKL inhibition suppressed tumor progression in a mouse model of lung adenocarcinoma. Therefore, denosumab may also be a therapeutic candidate for primary lung cancer in humans. In addition, the effect of female sex hormones on RANKL/RANK expression might explain how sex hormones are involved in lung cancer development.

### Multiple myeloma

Multiple myeloma is a malignant proliferative disease of plasma cells in the bone marrow and remains largely untreatable. Patients with multiple myeloma develop osteolytic lesions, which frequently lead to skeletal-related events, including hypercalcemia, spinal cord compression, and pathological fractures [105]. Therefore, preventing the progression of bone lesions is an important clinical issue in the treatment of myeloma. Myeloma-induced bone destruction is based on increased bone resorption and decreased bone formation, which are induced by the interaction between myeloma cells and the bone marrow microenvironment. Myeloma cells induce RANKL expression in stromal cells and suppress OPG expression. In a murine model of multiple myeloma, RANKL has been shown to induce myeloma cell release from dormancy through osteoclastic bone resorption, thereby promoting disease progression and/or relapse (Fig. 4c) [106]. Administration of RANK–Fc decreased tumor burden and the production of multiple myeloma-promoting cytokines such as IL-6. In line with these observations, bone resorption is related to tumor burden, and denosumab has been shown to prevent skeletal-related events in patients with multiple myeloma.

### Bone metastasis

Bone contains abundant growth factors, especially insulin-like growth factor (IGF) and transforming growth factor-β (TGF-β), which are continuously released into the bone marrow, together with the calcium that emerges through the bone resorption carried out by osteoclasts [2]. Under physiological conditions, these growth factors and calcium are utilized by osteoblasts to form new bone. On the other hand, when cancer cells metastasize to the bone marrow, these factors promote the growth and survival of cancer cells. Thus, bone provides a fertile environment for cancer cells.

Cancer cells that have metastasized to the bone marrow produce parathyroid hormone-related peptide (PTHrP) and other cytokines that stimulate RANKL expression and inhibit OPG expression in osteoblasts as well as stromal cells [107]. The increase in the RANKL/OPG ratio in the bone microenvironment leads to enhanced bone resorption and increased release of growth factors and calcium. These factors stimulate the further growth of cancer cells and the release of cancer cell-derived factors, thus increasing the RANKL/OPG ratio even more, thereby promoting continuous activation of bone destruction. This cascade of events is known as a “vicious cycle” that occurs between the growth of cancer cells and the destruction of bone (Fig. 4d) [107, 108]. The RANKL/RANK/OPG system is known to be involved in the development and metastasis of breast cancer, lung cancer, prostate cancer, melanoma, and renal cell carcinoma [99, 107]. In addition, the relative expression levels of RANKL, RANK, and OPG may have an influence on the prognosis of several cancer types, such as breast, lung, endometrial, renal cell, and gastric cancers, along with osteosarcoma and multiple myeloma [99]. Various studies have demonstrated a positive correlation between the level of RANK expression and the osteotropism of breast cancer and renal cell carcinoma. RANKL inhibition was shown to suppress the tumor burden in the bone in a mouse model of bone metastasis.

RANKL also regulates bone metastasis through the stimulation of the migration of cancer cells to bone [109]. A recent study showed that soluble RANKL is responsible for bone metastasis by promoting the migration of RANK-expressing tumor cells to bone without affecting bone resorption (Fig. 4d) [17]. RANKL/RANK signaling is also involved in both the induction of angiogenesis and increased vascular permeability via RANK-expressing endothelial cells, and may affect extravasation and metastasis (Fig. 4d) [110]. Indeed, the high level of serum RANKL is associated with an increased risk of developing bone metastasis in the patient with breast cancer [111].

Based on these findings, the RANKL/RANK axis plays a central role in various steps of bone metastasis. Therefore, inhibition of the RANKL/RANK pathway can break the vicious cycle and suppress bone metastasis [112]. Recently, it was reported that oral administration of AS2676293, a small-molecule inhibitor of RANKL, reduced bone metastasis of breast cancer cells and malignant melanoma by inhibiting not only bone resorption but also RANKL-induced tumor migration in a murine model [113].

## Conclusions

More than 20 years have passed since the discovery of RANKL, which was a major breakthrough in bone biology. RANKL was first identified in the immune system, and the sharing of this cytokine between bone metabolism and the immune system forms the basis for osteoimmunology. The studies that followed have revealed that RANKL plays a wide variety of roles in a variety of organs, sometimes beneficial and sometimes harmful. The findings that have accumulated as a result of these studies have established the richness of RANKL biology. More recently, the RANK–RANKL reverse signal was reported, which suggests that further development of the field of RANKL biology lies ahead. RANKL has been shown to be a good target for the treatment of osteoporosis, RA, and tumor. Additional studies may lead to the development of novel therapeutic strategies for yet other diseases.

## Abbreviations

Aire: Autoimmune regulator
Ang: Angiotensin
ARO: Autosomal recessive osteopetrosis
ATR: Angiotensin receptor
BBB: Blood–brain barrier
BMP: Bone morphogenetic protein
BRCA1: Breast cancer susceptibility gene 1
CCL20: C-C motif chemokine ligand 20
CCR6: C-C motif chemokine receptor 6
CD40L: CD40 ligand
CNS: Central nervous system
COX: Cyclooxygenase
DAMP: Damage-associated molecular pattern
DC: Dendritic cell
EP: Prostaglandin E receptor
ER: Estrogen receptor
ESH: Expansile skeletal hyperphosphatasia
FAE: Follicle-associated epithelium
FEO: Familial expansile osteolysis
GI tract: Gastrointestinal tract
HF: Hair follicle
HMGB1: High mobility group box-1
IFE: Interfollicular epidermis
IGF: Insulin-like growth factor
IH: Idiopathic hyperphosphatasia
IL: Interleukin
ILC: Innate lymphoid cell
iNKT cell: Invariant natural killer T cell
JPD: Juvenile Paget’s disease
LC: Langerhans cell
LN: Lymph node
LSn: Lateral septal nucleus
LTi: Lymphoid tissue inducer
LTo: Lymphoid tissue organizer
LTβR: Lymphotoxin β receptor
MaSC: Mammary stem cell
MHC: Major histocompatibility complex
MMTV: Mouse mammary tumor virus
MRC: Marginal reticular cell
MSn: Medial septal nucleus
mTEC: Medullary thymic epithelial cell
OCIF: Osteoclastogenesis inhibitory factor
ODF: Osteoclast differentiation factor
ODFR: ODF receptor
OPG: Osteoprotegerin
OPGL: OPG ligand
PDB2: Familial form of early-onset Paget’s disease of bone
PDL: Periodontal ligament
PGE2: Prostaglandin E2
POA: Preoptic area
PP: Peyer’s patch
PPARβ: Peroxisome proliferator-activated receptor β
Prx: Peroxiredoxin
PTHrP: Parathyroid hormone-related peptide
RA: Rheumatoid arthritis
RANK: Receptor activator of NF-κB
RANKL: receptor activator of NF-κB ligand
T2DM: Type 2 diabetes mellitus
TCR: T cell receptor
TGF-β: Transforming growth factor-β
T_H_17 cell: T helper 17 cell
TLR: Toll-like receptor
TNF: Tumor necrosis factor
TRANCE: TNF-related activation-induced cytokine
Treg cell: Regulatory T cell
TSA: Tissue-specific antigen
UV: Ultra violet
VSMC: Vascular smooth muscle cell

## References

[1] Nakashima T, Hayashi M, Takayanagi H (2012). New insights into osteoclastogenic signaling mechanisms. Trends Endocrinol Metab.

[2] Okamoto Kazuo, Nakashima Tomoki, Shinohara Masahiro, Negishi-Koga Takako, Komatsu Noriko, Terashima Asuka, Sawa Shinichiro, Nitta Takeshi, Takayanagi Hiroshi (2017). Osteoimmunology: The Conceptual Framework Unifying the Immune and Skeletal Systems. Physiological Reviews.

[3] Wong BR (1997). TRANCE is a novel ligand of the tumor necrosis factor receptor family that activates c-Jun N-terminal kinase in T cells. J Biol Chem.

[4] Anderson DM (1997). A homologue of the TNF receptor and its ligand enhance T-cell growth and dendritic-cell function. Nature.

[5] Kong YY (1999). OPGL is a key regulator of osteoclastogenesis, lymphocyte development and lymph-node organogenesis. Nature.

[6] Dougall WC (1999). RANK is essential for osteoclast and lymph node development. Genes Dev.

[7] Tsuda E (1997). Isolation of a novel cytokine from human fibroblasts that specifically inhibits osteoclastogenesis. Biochem Biophys Res Commun.

[8] Simonet WS (1997). Osteoprotegerin: a novel secreted protein involved in the regulation of bone density. Cell.

[9] Lacey DL (1998). Osteoprotegerin ligand is a cytokine that regulates osteoclast differentiation and activation. Cell.

[10] Yasuda H (1998). Osteoclast differentiation factor is a ligand for osteoprotegerin/osteoclastogenesis-inhibitory factor and is identical to TRANCE/RANKL. Proc Natl Acad Sci U S A.

[11] Nakagawa N (1998). RANK is the essential signaling receptor for osteoclast differentiation factor in osteoclastogenesis. Biochem Biophys Res Commun.

[12] Nelson CA, Warren JT, Wang MW, Teitelbaum SL, Fremont DH (2012). RANKL employs distinct binding modes to engage RANK and the osteoprotegerin decoy receptor. Structure.

[13] Nakashima Tomoki, Kobayashi Yasuhiro, Yamasaki Satoshi, Kawakami Atsushi, Eguchi Katsumi, Sasaki Hitoshi, Sakai Hideaki (2000). Protein Expression and Functional Difference of Membrane-Bound and Soluble Receptor Activator of NF-κB Ligand: Modulation of the Expression by Osteotropic Factors and Cytokines. Biochemical and Biophysical Research Communications.

[14] Nagashima K (2017). Identification of subepithelial mesenchymal cells that induce IgA and diversify gut microbiota. Nat Immunol.

[15] Tsukasaki M (2018). Host defense against oral microbiota by bone-damaging T cells. Nat Commun.

[16] Xiong J (2018). Soluble RANKL contributes to osteoclast formation in adult mice but not ovariectomy-induced bone loss. Nat Commun.

[17] Asano Tatsuo, Okamoto Kazuo, Nakai Yuta, Tsutsumi Masanori, Muro Ryunosuke, Suematsu Ayako, Hashimoto Kyoko, Okamura Tadashi, Ehata Shogo, Nitta Takeshi, Takayanagi Hiroshi (2019). Soluble RANKL is physiologically dispensable but accelerates tumour metastasis to bone. Nature Metabolism.

[18] Ikebuchi Y (2018). Coupling of bone resorption and formation by RANKL reverse signalling. Nature.

[19] Ono T, Nakashima T (2018). Recent advances in osteoclast biology. Histochem Cell Biol.

[20] Nakashima T (2011). Evidence for osteocyte regulation of bone homeostasis through RANKL expression. Nat Med.

[21] Xiong J (2011). Matrix-embedded cells control osteoclast formation. Nat Med.

[22] Xiong J (2015). Osteocytes, not osteoblasts or lining cells, are the main source of the RANKL required for osteoclast formation in remodeling bone. PLoS One.

[23] Sobacchi C (2007). Osteoclast-poor human osteopetrosis due to mutations in the gene encoding RANKL. Nat Genet.

[24] Guerrini Matteo M., Sobacchi Cristina, Cassani Barbara, Abinun Mario, Kilic Sara S., Pangrazio Alessandra, Moratto Daniele, Mazzolari Evelina, Clayton-Smith Jill, Orchard Paul, Coxon Fraser P., Helfrich Miep H., Crockett Julie C., Mellis David, Vellodi Ashok, Tezcan Ilhan, Notarangelo Luigi D., Rogers Michael J., Vezzoni Paolo, Villa Anna, Frattini Annalisa (2008). Human Osteoclast-Poor Osteopetrosis with Hypogammaglobulinemia due to TNFRSF11A (RANK) Mutations. The American Journal of Human Genetics.

[25] Nakatsuka Kiyoshi, Nishizawa Yoshiki, Ralston Stuart H (2003). Phenotypic Characterization of Early Onset Paget's Disease of Bone Caused by a 27-bp Duplication in the TNFRSF11A Gene. Journal of Bone and Mineral Research.

[26] Hughes Anne E., Ralston Stuart H., Marken John, Bell Christine, MacPherson Heather, Wallace Richard G.H., van Hul Wim, Whyte Michael P., Nakatsuka Kyoshi, Hovy Louis, Anderson Dirk M. (2000). Mutations in TNFRSF11A, affecting the signal peptide of RANK, cause familial expansile osteolysis. Nature Genetics.

[27] Whyte Michael P., Tau Cristina, McAlister William H., Zhang Xiafang, Novack Deborah V., Preliasco Virginia, Santini-Araujo Eduardo, Mumm Steven (2014). Juvenile Paget's disease with heterozygous duplication within TNFRSF11A encoding RANK. Bone.

[28] Palenzuela L (2002). Familial expansile osteolysis in a large Spanish kindred resulting from an insertion mutation in the TNFRSF11A gene. Journal of Medical Genetics.

[29] Johnson-Pais Teresa L, Singer Frederick R, Bone Henry G, McMurray Cynthia T, Hansen Marc F, Leach Robin J (2003). Identification of a Novel Tandem Duplication in Exon 1 of the TNFRSF11A Gene in Two Unrelated Patients With Familial Expansile Osteolysis. Journal of Bone and Mineral Research.

[30] Elahi Elahe, Shafaghati Yousef, Asadi Sareh, Absalan Farnaz, Goodarzi Hani, Gharaii Nava, Karimi-Nejad Mohammad Hassan, Shahram Farhad, Hughes Anne E. (2007). Intragenic SNP haplotypes associated with 84dup18 mutation in TNFRSF11A in four FEO pedigrees suggest three independent origins for this mutation. Journal of Bone and Mineral Metabolism.

[31] Whyte Michael P., Hughes Anne E. (2002). Expansile Skeletal Hyperphosphatasia Is Caused by a 15-Base Pair Tandem Duplication in TNFRSF11A Encoding RANK and Is Allelic to Familial Expansile Osteolysis. Journal of Bone and Mineral Research.

[32] Schafer AL (2014). Panostotic expansile bone disease with massive jaw tumor formation and a novel mutation in the signal peptide of RANK. J Bone Miner Res.

[33] Whyte MP (2002). Osteoprotegerin deficiency and juvenile Paget’s disease. N Engl J Med.

[34] Cundy T. (2002). A mutation in the gene TNFRSF11B encoding osteoprotegerin causes an idiopathic hyperphosphatasia phenotype. Human Molecular Genetics.

[35] Chong Belinda, Hegde Madhuri, Fawkner Matthew, Simonet Scott, Cassinelli Hamilton, Coker Mahmut, Kanis John, Seidel Joerg, Tau Cristina, Tüysüz Beyhan, Yüksel Bilgin, Love Donald, Cundy Tim (2003). Idiopathic Hyperphosphatasia andTNFRSF11BMutations: Relationships Between Phenotype and Genotype. Journal of Bone and Mineral Research.

[36] Whyte Michael P, Singhellakis Panagiotis N, Petersen Michael B, Davies Michael, Totty William G, Mumm Steven (2007). Juvenile Paget's Disease: The Second Reported, Oldest Patient Is Homozygous for the TNFRSF11B “Balkan” Mutation (966_969delTGACinsCTT), Which Elevates Circulating Immunoreactive Osteoprotegerin Levels. Journal of Bone and Mineral Research.

[37] Naot Dorit, Choi Ally, Musson David Shaun, Simsek Kiper Pelin Özlem, Utine Gulen Eda, Boduroglu Koray, Peacock Munro, DiMeglio Linda A., Cundy Tim (2014). Novel homozygous mutations in the osteoprotegerin gene TNFRSF11B in two unrelated patients with juvenile Paget's disease. Bone.

[38] Shoji-Matsunaga A, et al. Osteocyte regulation of orthodontic force-mediated tooth movement *via* RANKL expression. Sci Rep. 2017;7:8753. 10.1038/s41598-017-09326-7. 10.1038/s41598-017-09326-7 PMC5562866 28821826

[39] Compston Juliet E, McClung Michael R, Leslie William D (2019). Osteoporosis. The Lancet.

[40] Onal Melda, Xiong Jinhu, Chen Xinrong, Thostenson Jeff D., Almeida Maria, Manolagas Stavros C., O'Brien Charles A. (2012). Receptor Activator of Nuclear Factor κB Ligand (RANKL) Protein Expression by B Lymphocytes Contributes to Ovariectomy-induced Bone Loss. Journal of Biological Chemistry.

[41] Fujiwara Yuko, Piemontese Marilina, Liu Yu, Thostenson Jeff D., Xiong Jinhu, O'Brien Charles A. (2016). RANKL (Receptor Activator of NFκB Ligand) Produced by Osteocytes Is Required for the Increase in B Cells and Bone Loss Caused by Estrogen Deficiency in Mice. Journal of Biological Chemistry.

[42] Kawai M, Modder UI, Khosla S, Rosen CJ (2011). Emerging therapeutic opportunities for skeletal restoration. Nat Rev Drug Discov.

[43] Fukumoto S, Matsumoto T (2017). Recent advances in the management of osteoporosis. F1000Res.

[44] Mullard A (2019). FDA approves first-in-class osteoporosis drug. Nat Rev Drug Discov.

[45] Wijenayaka AR (2011). Sclerostin stimulates osteocyte support of osteoclast activity by a RANKL-dependent pathway. PLoS One.

[46] Tu Xiaolin, Delgado-Calle Jesus, Condon Keith W., Maycas Marta, Zhang Huajia, Carlesso Nadia, Taketo Makoto M., Burr David B., Plotkin Lilian I., Bellido Teresita (2015). Osteocytes mediate the anabolic actions of canonical Wnt/β-catenin signaling in bone. Proceedings of the National Academy of Sciences.

[47] Takayanagi H (2007). Osteoimmunology: shared mechanisms and crosstalk between the immune and bone systems. Nat Rev Immunol.

[48] Danks L (2016). RANKL expressed on synovial fibroblasts is primarily responsible for bone erosions during joint inflammation. Ann Rheum Dis.

[49] Sato K (2006). Th17 functions as an osteoclastogenic helper T cell subset that links T cell activation and bone destruction. J Exp Med.

[50] Komatsu Noriko, Okamoto Kazuo, Sawa Shinichiro, Nakashima Tomoki, Oh-hora Masatsugu, Kodama Tatsuhiko, Tanaka Sakae, Bluestone Jeffrey A, Takayanagi Hiroshi (2013). Pathogenic conversion of Foxp3+ T cells into TH17 cells in autoimmune arthritis. Nature Medicine.

[51] Takeuchi T (2019). Effects of the anti-RANKL antibody denosumab on joint structural damage in patients with rheumatoid arthritis treated with conventional synthetic disease-modifying antirheumatic drugs (DESIRABLE study): a randomised, double-blind, placebo-controlled phase 3 trial. Ann Rheum Dis.

[52] Tanaka Sakae (2019). RANKL is a therapeutic target of bone destruction in rheumatoid arthritis. F1000Research.

[53] Ono T. Why and how do teeth come off? -New insights into the tooth loss during periodontitis-. Dent Oral Craniofac Res. 2018;4. 10.15761/docr.1000259.

[54] Tsukasaki Masayuki, Takayanagi Hiroshi (2019). Osteoimmunology: evolving concepts in bone–immune interactions in health and disease. Nature Reviews Immunology.

[55] Penna S, Capo V, Palagano E, Sobacchi C, Villa A. One Disease, Many Genes: Implications for the Treatment of Osteopetroses. Front Endocrinol. 2019;10:85. 10.3389/fendo.2019.00085. 10.3389/fendo.2019.00085 PMC6389615 30837952

[56] Coudert AE, de Vernejoul MC, Muraca M, Del Fattore A (2015). Osteopetrosis and its relevance for the discovery of new functions associated with the skeleton. Int J Endocrinol.

[57] Inglesfield S, Cosway EJ, Jenkinson WE, Anderson G (2019). Rethinking Thymic Tolerance: Lessons from Mice. Trends Immunol.

[58] Akiyama T, Shinzawa M, Akiyama N (2012). RANKL-RANK interaction in immune regulatory systems. World J Orthop.

[59] Anderson MS, Su MA (2016). AIRE expands: new roles in immune tolerance and beyond. Nat Rev Immunol.

[60] Rossi Simona W., Kim Mi-Yeon, Leibbrandt Andreas, Parnell Sonia M., Jenkinson William E., Glanville Stephanie H., McConnell Fiona M., Scott Hamish S., Penninger Josef M., Jenkinson Eric J., Lane Peter J.L., Anderson Graham (2007). RANK signals from CD4+3− inducer cells regulate development of Aire-expressing epithelial cells in the thymic medulla. The Journal of Experimental Medicine.

[61] Hikosaka Y (2008). The cytokine RANKL produced by positively selected thymocytes fosters medullary thymic epithelial cells that express autoimmune regulator. Immunity.

[62] Roberts Natalie A., White Andrea J., Jenkinson William E., Turchinovich Gleb, Nakamura Kyoko, Withers David R., McConnell Fiona M., Desanti Guillaume E., Benezech Cecile, Parnell Sonia M., Cunningham Adam F., Paolino Magdalena, Penninger Josef M., Simon Anna Katharina, Nitta Takeshi, Ohigashi Izumi, Takahama Yousuke, Caamano Jorge H., Hayday Adrian C., Lane Peter J.L., Jenkinson Eric J., Anderson Graham (2012). Rank Signaling Links the Development of Invariant γδ T Cell Progenitors and Aire+ Medullary Epithelium. Immunity.

[63] White AJ (2014). An essential role for medullary thymic epithelial cells during the intrathymic development of invariant NKT cells. J Immunol.

[64] Chang JE, Turley SJ (2015). Stromal infrastructure of the lymph node and coordination of immunity. Trends Immunol.

[65] Katakai T (2012). Marginal reticular cells: a stromal subset directly descended from the lymphoid tissue organizer. Front Immunol.

[66] Mueller CG, Hess E (2012). Emerging functions of RANKL in lymphoid tissues. Front Immunol.

[67] Vondenhoff Mark F., Greuter Mascha, Goverse Gera, Elewaut Dirk, Dewint Pieter, Ware Carl F., Hoorweg Kerim, Kraal Georg, Mebius Reina E. (2009). LTβR Signaling Induces Cytokine Expression and Up-Regulates Lymphangiogenic Factors in Lymph Node Anlagen. The Journal of Immunology.

[68] Camara A (2019). Lymph node mesenchymal and endothelial stromal cells cooperate via the RANK-RANKL cytokine axis to shape the sinusoidal macrophage niche. Immunity.

[69] Spits H (2013). Innate lymphoid cells—a proposal for uniform nomenclature. Nat Rev Immunol.

[70] Panda SK, Colonna M (2019). Innate lymphoid cells in mucosal immunity. Front Immunol.

[71] Sawa Shinichiro, Cherrier Marie, Lochner Matthias, Satoh-Takayama Naoko, Fehling Hans Jörg, Langa Francina, Di Santo James P., Eberl Gérard (2010). Lineage Relationship Analysis of RORγt+Innate Lymphoid Cells. Science.

[72] Sawa Shinichiro, Lochner Matthias, Satoh-Takayama Naoko, Dulauroy Sophie, Bérard Marion, Kleinschek Melanie, Cua Daniel, Di Santo James P, Eberl Gérard (2011). RORγt+ innate lymphoid cells regulate intestinal homeostasis by integrating negative signals from the symbiotic microbiota. Nature Immunology.

[73] Bando JK (2018). The tumor necrosis factor superfamily member RANKL suppresses effector cytokine production in group 3 innate lymphoid cells. Immunity.

[74] Lugering A (2010). CCR6 identifies lymphoid tissue inducer cells within cryptopatches. Clin Exp Immunol.

[75] Ohno H (2016). Intestinal M cells. J Biochem.

[76] Knoop KA (2009). RANKL is necessary and sufficient to initiate development of antigen-sampling M cells in the intestinal epithelium. J Immunol.

[77] Clayton K, Vallejo AF, Davies J, Sirvent S, Polak ME (2017). Langerhans Cells-Programmed by the Epidermis. Front Immunol.

[78] Honda T, Egawa G, Kabashima K (2019). Antigen presentation and adaptive immune responses in skin. Int Immunol.

[79] Soontrapa K (2011). Prostaglandin E2-prostaglandin E receptor subtype 4 (EP4) signaling mediates UV irradiation-induced systemic immunosuppression. Proc Natl Acad Sci U S A.

[80] Loser K (2006). Epidermal RANKL controls regulatory T-cell numbers via activation of dendritic cells. Nat Med.

[81] Hart PH, Norval M (2018). Ultraviolet radiation-induced immunosuppression and its relevance for skin carcinogenesis. Photochem Photobiol Sci.

[82] Forrester JV, McMenamin PG, Dando SJ (2018). CNS infection and immune privilege. Nat Rev Neurosci.

[83] Guerrini MM (2015). Inhibition of the TNF family cytokine RANKL prevents autoimmune inflammation in the central nervous system. Immunity.

[84] Shimamura M (2014). OPG/RANKL/RANK axis is a critical inflammatory signaling system in ischemic brain in mice. Proc Natl Acad Sci U S A.

[85] Shichita Takashi, Sugiyama Yuki, Ooboshi Hiroaki, Sugimori Hiroshi, Nakagawa Ryusuke, Takada Ichiro, Iwaki Toru, Okada Yasunori, Iida Mitsuo, Cua Daniel J, Iwakura Yoichiro, Yoshimura Akihiko (2009). Pivotal role of cerebral interleukin-17–producing γδT cells in the delayed phase of ischemic brain injury. Nature Medicine.

[86] Shichita T (2012). Peroxiredoxin family proteins are key initiators of post-ischemic inflammation in the brain. Nat Med.

[87] Bonaventura Aldo, Liberale Luca, Vecchié Alessandra, Casula Matteo, Carbone Federico, Dallegri Franco, Montecucco Fabrizio (2016). Update on Inflammatory Biomarkers and Treatments in Ischemic Stroke. International Journal of Molecular Sciences.

[88] Fata JE (2000). The osteoclast differentiation factor osteoprotegerin-ligand is essential for mammary gland development. Cell.

[89] Cao Yixue, Bonizzi Giuseppina, Seagroves Tiffany N., Greten Florian R., Johnson Randall, Schmidt Emmett V., Karin Michael (2001). IKKα Provides an Essential Link between RANK Signaling and Cyclin D1 Expression during Mammary Gland Development. Cell.

[90] Rao S, Cronin SJF, Sigl V, Penninger JM (2018). RANKL and RANK: From Mammalian Physiology to Cancer Treatment. Trends Cell Biol.

[91] Hanada R (2009). Central control of fever and female body temperature by RANKL/RANK. Nature.

[92] Panizo S (2009). RANKL increases vascular smooth muscle cell calcification through a RANK-BMP4-dependent pathway. Circ Res.

[93] Osako MK (2010). Estrogen inhibits vascular calcification via vascular RANKL system: common mechanism of osteoporosis and vascular calcification. Circ Res.

[94] Osako Mariana Kiomy, Nakagami Hironori, Shimamura Munehisa, Koriyama Hiroshi, Nakagami Futoshi, Shimizu Hideo, Miyake Takashi, Yoshizumi Masao, Rakugi Hiromi, Morishita Ryuichi (2013). Cross-Talk of Receptor Activator of Nuclear Factor-κB Ligand Signaling With Renin–Angiotensin System in Vascular Calcification. Arteriosclerosis, Thrombosis, and Vascular Biology.

[95] Duheron V., Hess E., Duval M., Decossas M., Castaneda B., Klopper J. E., Amoasii L., Barbaroux J.-B., Williams I. R., Yagita H., Penninger J., Choi Y., Lezot F., Groves R., Paus R., Mueller C. G. (2011). Receptor activator of NF- B (RANK) stimulates the proliferation of epithelial cells of the epidermo-pilosebaceous unit. Proceedings of the National Academy of Sciences.

[96] Kiechl Stefan, Wittmann Jürgen, Giaccari Andrea, Knoflach Michael, Willeit Peter, Bozec Aline, Moschen Alexander R, Muscogiuri Giovanna, Sorice Gian Pio, Kireva Trayana, Summerer Monika, Wirtz Stefan, Luther Julia, Mielenz Dirk, Billmeier Ulrike, Egger Georg, Mayr Agnes, Oberhollenzer Friedrich, Kronenberg Florian, Orthofer Michael, Penninger Josef M, Meigs James B, Bonora Enzo, Tilg Herbert, Willeit Johann, Schett Georg (2013). Blockade of receptor activator of nuclear factor-κB (RANKL) signaling improves hepatic insulin resistance and prevents development of diabetes mellitus. Nature Medicine.

[97] Dufresne Sébastien S., Dumont Nicolas A., Boulanger-Piette Antoine, Fajardo Val A., Gamu Daniel, Kake-Guena Sandrine-Aurélie, David Rares Ovidiu, Bouchard Patrice, Lavergne Éliane, Penninger Josef M., Pape Paul C., Tupling A. Russell, Frenette Jérôme (2016). Muscle RANK is a key regulator of Ca2+ storage, SERCA activity, and function of fast-twitch skeletal muscles. American Journal of Physiology-Cell Physiology.

[98] Bonnet Nicolas, Bourgoin Lucie, Biver Emmanuel, Douni Eleni, Ferrari Serge (2019). RANKL inhibition improves muscle strength and insulin sensitivity and restores bone mass. Journal of Clinical Investigation.

[99] Peters S, Clezardin P, Marquez-Rodas I, Niepel D, Gedye C (2019). The RANK-RANKL axis: an opportunity for drug repurposing in cancer?. Clin Transl Oncol.

[100] Gonzalez-Suarez E (2010). RANK ligand mediates progestin-induced mammary epithelial proliferation and carcinogenesis. Nature.

[101] Schramek D (2010). Osteoclast differentiation factor RANKL controls development of progestin-driven mammary cancer. Nature.

[102] Nolan E (2016). RANK ligand as a potential target for breast cancer prevention in BRCA1-mutation carriers. Nat Med.

[103] Sigl V (2016). RANKL/RANK control Brca1 mutation. Cell Res.

[104] Rao S (2017). RANK rewires energy homeostasis in lung cancer cells and drives primary lung cancer. Genes Dev.

[105] Raje NS, Bhatta S, Terpos E (2019). Role of the RANK/RANKL Pathway in Multiple Myeloma. Clin Cancer Res.

[106] Lawson MA (2015). Osteoclasts control reactivation of dormant myeloma cells by remodelling the endosteal niche. Nat Commun.

[107] de Groot AF, Appelman-Dijkstra NM, van der Burg SH, Kroep JR (2018). The anti-tumor effect of RANKL inhibition in malignant solid tumors - A systematic review. Cancer Treat Rev.

[108] Capietto AH, Faccio R. Immune regulation of bone metastasis. Bonekey Rep. 2014;3:600. 10.1038/bonekey.2014.95. 10.1038/bonekey.2014.95 PMC4260446 25512853

[109] Jones DH (2006). Regulation of cancer cell migration and bone metastasis by RANKL. Nature.

[110] Renema N, Navet B, Heymann MF, Lezot F, Heymann D. RANK-RANKL signalling in cancer. Biosci Rep. 2016;36. 10.1042/bsr20160150. 10.1042/BSR20160150 PMC4974605 27279652

[111] Rachner TD (2019). Prognostic value of RANKL/OPG serum levels and disseminated tumor cells in nonmetastatic breast cancer. Clin Cancer Res.

[112] Celia-Terrassa T, Kang Y (2018). Metastatic niche functions and therapeutic opportunities. Nat Cell Biol.

[113] Nakai Y (2019). Efficacy of an orally active small-molecule inhibitor of RANKL in bone metastasis. Bone Res.

